# Effects of external irradiation of the neck region on intima media thickness of the common carotid artery

**DOI:** 10.1186/1476-7120-8-8

**Published:** 2010-03-19

**Authors:** Maria Elena Gianicolo, Emilio Antonio Luca Gianicolo, Francesco Tramacere, Maria Grazia Andreassi, Maurizio Portaluri

**Affiliations:** 1 ISBEM Euro Mediterranean Scientific Biomedical Institute, Brindisi, Italy; 2 CNR Institute of Clinical Physiology, Lecce, Italy; 3 Radiotherapy Dept. ASL Brindisi, Ospedale Perrino, Brindisi, Italy; 4 CNR Institute of Clinical Physiology, Pisa, Italy

## Abstract

**Background:**

Several studies have shown that common carotid intima-media thickness (IMT) is increased after radiotherapy (RT) to the head and neck. However, further studies are needed to define the exact mechanism of radiation-induced injury in large vessels, investigate the relationship between radiation dose and large vessel injury and evaluate the rate of progress of atherosclerosis in irradiated vessels.

**Objectives:**

To investigate whether external irradiation to the carotid area has any effect on IMT of the common carotid artery in a group of patients who received RT vs control group matched for age, gender and race.

**Methods:**

We studied 19 patients (10 male; 47.8 ± 17.4 years) during a 5-month period (January 2009-July 2009); they had completed RT with a mean of 2.9 years before (range: 1 month-6 years) The mean radiation dose to the neck in the irradiated patients was 41.2 ± 15.6 Gy (range: 25-70 Gy). Common carotid IMT was measured with echo-color Doppler. Nineteen healthy adult patients (10 male; 47.8 ± 17.6) were recruited as a control group.

**Results:**

IMT was not significantly higher in patients when compared to the control group (0.59 ± 0.16 vs 0.56 ± 0.16 mm, p = 0.4). There was no significant difference between the two groups in relation to the absence (p = 0.7) or presence (p = 0.6) of vascular risk factors. Although the difference did not reach statistical significance (p = 0.1), the irradiated young patients (age ≤ 52 years) had IMT measurements higher (0.54 ± 0.08 mm) than the non-irradiated young patients (0.49 ± 0.14 mm). The mean carotid IMT increased with increasing doses of radiation to the neck (p = 0.04).

**Conclusion:**

This study shows that increased IMT of the common carotid artery after RT is radiation-dose-related. Therefore it is important to monitor IMT, which can be used as an imaging biomarker for early diagnosis of cerebrovascular disease in patients who have had radiotherapy for treatment of cancer of the head and neck and who are at increased risk for accelerated atherosclerosis in carotid arteries.

## Introduction

Radiotherapy (RT) as a single modality or in combination with surgery has been widely used in the treatment of head and neck tumours for many years, and this has resulted in a marked improvement in survival of patients with these tumours, who previously had a dismal prognosis. Successful treatment increases survival but also puts the patient at risk of radiation-related side effects. Of these, vascular side effects are serious and may be life-threatening.

RT involving the head and neck or supraclavicular (SC) region necessarily include segments of the carotid artery. It has traditionally been accepted that the carotid is fairly resistant to the fibrosis and narrowing that are evident in smaller vessels undergoing comparable radiation exposure [1,2]. However, several papers have described increasing frequency of stroke or transient ischemic attack after RT [3-8]. Increased common carotid intima-media thickness assessed by high frequency ultrasound is an early marker of atherosclerosis and a predictor of subsequent death from myocardial infarction and stroke [9].

Indeed, recent studies have shown that common carotid IMT is increased after RT to the head and neck [10-12]. However, the clinical relevance of these observations remains unknown, and further studies are essential to define the role of IMT as a non-invasive diagnostic tool for identifying subclinical vascular disease and estimating risk of future cardiovascular disease in long-term cancer survivors. Therefore, the aim of this study was to examine IMT in patients who received irradiation to the carotid arteries during radiotherapy vs. control group matched for age, gender and race, by using B-mode ultrasonography.

## Patients and Methods

### Patient population

Over a 5-month period (January 2009-July 2009), we studied nineteen asymptomatic patients (47.8 ± 17.4 years) who had completed RT. Only patients who received radiation doses to the neck were selected. They were recruited from the follow-up activity of the Radiotherapy Division of "Perrino" Hospital, Brindisi, and were sent to the ultrasound laboratory at the Division of Cardiology of the same hospital. All the patients were studied for the common risk factors for atherosclerosis.

Ten patients were males (42.3 ± 15.9 years) and nine patients were women (54 ± 17.8 years). More than half of the patients (58%) were < 52 years old. Nine patients received RT symmetrically, on the left and right carotid.

Nineteen healthy adult patients (10 male; 47.8 ± 17.6) were recruited for the control group. They had no history of irradiation to the head and neck. Age, sex and race of the control group matched those of the irradiated group, while risk factors not were matched individually, but for frequency.

The risk factors were studied individually or by using a synthetic indicator, which analyzes the frequency of any risk factor or at least one risk factor in irradiated patients and in the control group.

None of the patients had a history of previous stroke or transient ischemic attack. Furthermore, patients with carotid plaques were excluded from the study for both groups. The maximum time elapsed between radiotherapy and ultrasound examination was 6 years, and the minimum time was 1 month. A complete history, including cardiovascular risk factors such as hypertension, diabetes, and dyslipidemia, was collected from all patients. Diabetes mellitus [13], arterial hypertension [14], hypertriglyceridemia and hypercholesterolemia [15]http://www.nhlbi.nih.gov/guidelines/cholestrol/index.htm.were defined according to standard definitions.

Current smokers were defined as patients with moderate or heavy cigarette use (at least 3 cigarettes per day) at the time of entry into the study. Former smokers were defined as the participants who had smoked regularly, at least 3 cigarettes/day, and who had quit smoking for at least 6 months at the time of enrolment. Non-smokers were defined as patients who had never smoked before admission. Smoker patients were the combined group of the past and current smokers. Accordingly, each risk factor was coded as either present or absent.

### Carotid measurements

Carotid IMT of the arterial wall was determined using a Philips IE33 Ultrasound at high resolution, with 5-12 MHz linear array transducer. Patients were placed comfortably in the supine position with the head directed away from the side of interest and the neck extended slightly. Both common carotid arteries were examined along their full visible length. All measurements of IMT were made in the longitudinal plane at the point of maximum thickness on the far wall of the common carotid artery.

The carotid IMT assessment was supplemented by a thorough scan of the extracranial carotid arteries for the eventual presence of carotid plaques, to increase sensitivity for identifying subclinical vascular disease. The mean intima-media thickness was calculated using the semiautomated edge-detection software package (QLAB) 1 cm distant from the carotid arterial bifurcation. This is a program incorporated in the software package of the ultrasound equipment.

### Statistical analysis

Data are expressed as means ± standard deviation. Differences in non-continuous variables were tested by χ^2 ^analysis. Differences between the means of the two continuous variables were evaluated by Student's *t*-test. Regression analysis with Pearson's test was also used to evaluate the relationship between the two continuous variables. Multiple regression analyses were carried out with IMT values as dependent variables, and relevant parameters (radiation dose, age, vascular risk factors) as independent variables. The level of significance set at p < 0.05 was considered for all statistical analyses. Statistical analysis was performed using SAS statistical software (SAS Institute Inc, Cary, NC) versions 8.2 per Microsoft Windows.

## Results

The demographic and clinical characteristics of the study population are reported in Table 1. Six patients in the control group did not present any risk factor (31.6%) and 13 patients had at least one risk factor (68.4%); five irradiated patients (26.3%) did not present any risk factor and fourteen patients (73.7%) had at least one risk factor.

**Table 1:** Demographic and clinical characteristics of the study population

Demographic characteristics/Risk factors	Patient group (n = 19)	Control group (n = 19)
Age, years (mean ± SD)	47.8 ± 17.4	47.8 ± 17.6
Max	22	22
Min	71	73
*≤ 52 years*		
Number of Subjects (%)	11 (57.9)	11 (57.9)
*≥ 53 years*		
Number of Subjects (%)	8 (42.1)	8 (42.1)
		
Sex		
*Female*		
Number of Subjects	9 (47.4)	9 (47.4)
Age, years (mean ± SD)	54 ± 17.8	54.3 ± 18.1
Max	23	23
Min	71	73
*Male*		
Number of Subjects	10 (52.6)	10 (52.6)
Age, years (mean ± SD)	42.3 ± 15.9	42 ± 15.9
Max	22	22
Min	71	70
		
Risk factors (%)		
*No risk factor*	5 (26.3)	6 (31.6)
*At least one risk factor*	14 (73.7)	13 (68.4)
*Current smoker*	4 (21)	4 (21)
*Smoking past*	3 (15.8)	3 (15.8)
*Hypertension*	4 (21)	5 (26.3)
*Hypercholesterolemia*	4 (21)	2 (10.5)
*Hypertriglyceridemia*	2 (10.5)	3 (15.8)
*Family history of cardiovascular and cerebrovascular disease*	5 (26.3)	8 (42.1)
*Diabetes mellitus*	1 (5.3)	3 (15.8)
*Overweight/Obesity*	10 (52.6)	8 (42.1)
Therapy (%)		
*No therapy*	15 (79%)	14 (73.7%)
*Antidiabetic therapy*	1 (5.2%)	0
*Lipid-lowering drugs*	2 (10.5%)	1 (5.2%)
*Anti-hypertensive drugs*	4 (21%)	5 (26.3%)

Hypercholesterolemia was not significantly higher (21.1%) in irradiated patients than in control patients (10.5%, p = 0.37). Family history of cardiovascular or cerebrovascular diseases was higher in control patients (42.1%, p = 0.31) than in irradiated patients (26.3%), and diabetes was more frequent in control patients (15.5%) than in irradiated patients (5.3%, p = 0.29).

Over 70% of patients the control group and irradiated receiving no therapy and more than 20% of patients in both groups taking antihypertensive drugs.

Tumor type and RT dose are summarized in Table 2. All the patients received radiation doses to the neck in the range 25-70 Gy. The mean radiation dose to the neck in the irradiated patients was 41.2 ± 15.6 Gy. The median post-RT time was 1046 days (range 29-2492 days).

**Table 2:** Tumor characteristics and radiation dose

Clinical Characteristics	Number of patients (%)	Mean dose (Gy) ± SD	Max	Min
Tumor Type				
*H Lymphoma*	10 (52.6)	-	-	-
*NH Lymphoma*	1 (5.3)	-	-	-
*Nasopharyngeal carcinoma*	2 (10.5)	-	-	-
*Oral cavity carcinoma*	1 (5.3)	-	-	-
*Oropharyngeal carcinoma*	1 (5.3)	-	-	-
*Laryngeal carcinoma*	2 (10.5)	-	-	-
*Breast carcinoma*	2 (10.5)	-	-	-
Dose RT to the neck	19	41.2 ± 15.6	70	25.2
*Female*	9 (47.4)	44.7 ± 13.2	30	60
*Male*	10 (52.6)	38.1 ± 15.7	70	25.2
*≤ 52 years*	11 (58.0)	31.2 ± 8.8	25.2	56
*≥ 53 years*	8 (42.0)	55.0 ± 12.2	30	70

H = Hodgkin's

NH = non-Hodgkin's

RT = radiotherapy

Gy = Gray

We also observed a higher IMT in males (0.61 ± 0.19 mm) compared to females (0.57 ± 0.13 mm) for irradiated carotid (p = 0.56). However, non-irradiated carotid IMT was higher in females (0.61 ± 0.16 mm) than in males (0.51 ± 0.15 mm) (p = 0.06). We did not find a significant association between carotid IMT and age in patient groups (p = 0.06); while the association was statistically significant in the control group (p < 0.0001).

**Figure 1:**
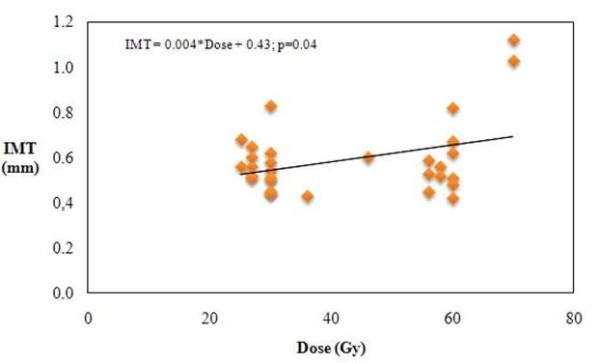
**Linear regression between IMT and RT dose**.

IMT measurements of the irradiated carotid were not significantly higher in patients compared to control group (Table 3). There was no significant difference between the two groups in relation to the absence (p = 0.7) or presence (p = 0.6) of vascular risk factors. The irradiated young patients (age = 52 years) had IMT measurements higher (0.54 ± 0.08 mm) than the non-irradiated young patients (0.49 ± 0.14 mm). The difference was not statistically significant (p = 0.1), but suggested the existence of a relationship (Table 3). The mean carotid IMT increases with increasing radiation dose to the neck (p = 0.04) (Fig. 1). There was no linear correlation between the IMT and the post-RT time.

**Table 3:** IMT measurement in irradiated and non-irradiated patients

Groups	No. of measurements	Mean (mm)	SD	Maximum (mm)	Minimum (mm)	*t*-test p-value
All subjects						
*Irradiated*	31	0.59	0.16	1.12	0.42	0.42
*Non-irradiated*	38	0.56	0.16	0.97	0	
						
Sex						
Female						
*Irradiated*	14	0.57	0.13	0.83	0.43	0.55
*Not-irradiated*	18	0.61	0.16	0.97	0.42	
Male						
*Irradiated*	17	0.61	0.19	1.12	0.42	0.1
*Non-irradiated*	20	0.51	0.15	0.79	0	
						
Age						
≤ 52 years						
*Irradiated*	17	0.54	0.08	0.68	0.43	0.14
*Non-irradiated*	22	0.49	0.14	0.79	0	
≥ 53 years						
*Irradiated*	14	0.65	0.22	1.12	0.42	0.92
*Non-irradiated*	16	0.66	0.13	0.97	0.52	
						
Risk factors						
No risk factors						
*Irradiated*	6	0.54	0.09	0.68	0.43	0.73
*Non-irradiated*	12	0.50	0.25	0.97	0	
At least a risk factor						
*Irradiated*	25	0.60	0.18	1.12	0.42	0.64
*Non-irradiated*	26	0.59	0.09	0.79	0.43	

## Discussion

This study shows that the IMT of the common carotid artery increases with increasing doses of radiation to the neck. Moreover, it is noteworthy that in our study a significant association with age was found only in healthy adult controls and not in cancer survivors. Interestingly, the irradiated young patients showed higher IMT measurements than the non-irradiated young patients.

Our findings are in agreement with previous observations. In a previous study, the median low cervical radiation dose was 38 Gy (range, 30-57 Gy) for those who developed carotid artery disease [16]. The minimal dose for RT damage of the cervical arteries was assumed to be 41 Gy [17]. A recent study has shown that there were two distinct subgroups of Hodgkin lymphoma survivors who developed non-coronary atherosclerotic vascular disease. The first group is an older population with probable pre-existing disease either unaffected or only accelerated by radiation. These patients experienced strokes and TIAs, were older at RT exposure (median age, 51 years), and had a relatively short time interval (median, 5.6 years) from RT to development of vascular disease. The second group differs in that the patients were younger (median, 20 years) at RT exposure, and had a longer latency period before diagnosis (median, 20.8 years) [16].

Radiotherapy to the neck is believed to predispose to atherosclerosis. Recently, irradiation of the neck has been associated with subsequent vascular wall thickening [18], accelerated atherosclerotic plaque formation [19], decreased flow on ultrasound [20], and coronary artery stenosis [21]. A recent study found that irradiation of the neck of Hodgkin (H) and non-Hodgkin (NH) patients determines a greater IMT of the arterial wall than in controls matched for the classic atherosclerosis risk factors, so authors concluded that radiotherapy can influence the atherosclerotic process [22,23].

On the other hand, some researchers have documented no increase in clinical cerebrovascular events (CVEs) after SC RT [24,25], in breast cancer patients.

The exact mechanism of radiation injury remains uncertain. Injury to the vasa vasorum and consequent ischemic lesions of the arterial wall were thought to be morphological features distinguishing radiation-induced arterial injury from spontaneous atherosclerosis [26-29].

Many factors have been associated with increased risk of developing atherosclerosis--for example, increasing age, diabetes mellitus, hypertension, hypercholesterolemia, smoking and irradiation to the affected vessels [30].

It has been assumed that the carotid lesion in an irradiated artery arises from an accelerated atherosclerotic process, and the patient's subsequent risk of stroke would be similar to those who did not receive radiation but with an equivalent degree of luminal stenosis [31,32].

The potential for stroke is well-recognized in patients with head and neck cancer and is generally considered a risk related to pre-existing atherosclerotic disease. Since the IMT of the common carotid artery is a good predictor of stroke [33], data from other reports should heighten awareness that patients who have had radiotherapy for treatment of head and neck malignancy are at increased risk of accelerated atherosclerosis in carotid arteries, which in turn can lead to cerebrovascular accidents. As most patients with early changes in post-radiation carotid injury remain asymptomatic, early detection and monitoring are possible by routine ultrasound examination and measurement of the IMT of common carotid arteries.

Hypertension, diabetes mellitus, hypercholesterolemia and obesity are known to exacerbate the severity and speed of atherosclerosis. Effective management of these modifiable factors with appropriate changes in lifestyle may halt or slow the development of severe atherosclerosis leading to carotid stenosis or cerebrovascular events. Meanwhile, we recommend a higher level of alert and routine surveillance with ultrasonography in all patients with carotid artery stenosis induced by radiation therapy. However, recommendation of a routine exam certainly does not derive from our small number of patients, but is based on several previously-mentioned studies.

The study limitations were the small number of patients, the variable time interval from RT to vascular ultrasound, and lack of pre-RT baseline. Another restriction is the lack of follow-up in one patient. Additional studies are needed on a larger number of patients for early detection of the IMT changes as a predictive sign of atherosclerotic risk in irradiated patients.

The most important clinical implication is establishing appropriateness of indications for radiotherapy to the neck, especially in younger patients.

## Competing interests

The authors declare that they have no competing interests.

## Authors' contributions

MEG performed the carotid Doppler examinations and drafting of the manuscript; EAG participated in data collection and performed the statistical analysis; FT partecipated in patient recruitment and in data collection; MGA participated in the statistical analysis of the results and critically revised the manuscript; MP played a main role in the design and coordination of the study, and in writing of the manuscript. All authors read and approved the final manuscript.
